# 2,4-Diphenyl-6-trifluoro­methyl-2,3-dihydro-1*H*,5*H*-pyrrolo­[3,4-*c*]pyrrole-1,3-dione

**DOI:** 10.1107/S1600536812001675

**Published:** 2012-01-21

**Authors:** Sue A. Roberts, Guillermo Martinez-Ariza, Justin Dietrich, Christopher Hulme

**Affiliations:** a Department of Chemistry and Biochemistry, 1041 E. Lowell St, The University of Arizona, Tucson, AZ 85721, USA; b Departamento de Quimica, Division de Ciencias Naturales y Exactas, Universidad de Guanajuato, Col. Noria Alta s/n C.P. 36050, Guanajuato, Gto., Mexico; c Southwest Center for Drug Discovery and Development, College of Pharmacy, BIO5 Institute, The University of Arizona, Tucson, AZ 85721, USA

## Abstract

The asymmetric unit of the title compound, C_19_H_11_F_3_N_2_O_2_, contains two crystallographically unique mol­ecules which differ in the rotation of a phenyl ring and a –CF_3_ substituent. The dihedral angles involving the pyrrole ring and the attached phenyl ring are 62.82 (8) and 71.54 (7)° in the two molecules. The difference in the rotation of the CF_3_ groups with respect to the pyrrolo rings to which they are attached is 23.5(1)°. For one mol­ecule, there is a close contact between an H atom and the centroid of the phenyl ring of an adjacent mol­ecule (2.572 Å). A similar contact is lacking in the second mol­ecule. In the crystal, N—H⋯O inter­actions connect adjacent mol­ecules into a chain normal to (01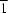). Crystallographically unique mol­ecules alternate along the hydrogen-bonded chains.

## Related literature

For background information on the biological activity of compounds with pyrrol-3,4-dicarboximide scaffolds, see: Malinka *et al.* (1999 ▶, 2005 ▶); Shen *et al.* (2010 ▶). For a description of structurally similar lamellarins, see: Yu *et al.* (2011 ▶).
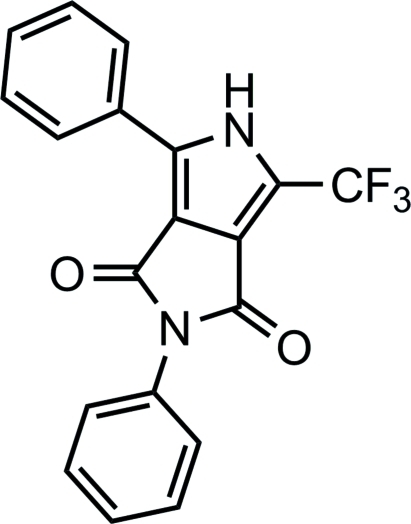


## Experimental

### 

#### Crystal data

C_19_H_11_F_3_N_2_O_2_
*M* *_r_* = 356.30
Triclinic, 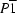

*a* = 10.4730 (4) Å
*b* = 12.2394 (5) Å
*c* = 13.4379 (5) Å
α = 67.542 (2)°
β = 82.511 (2)°
γ = 80.294 (2)°
*V* = 1564.98 (11) Å^3^
*Z* = 4
Mo *K*α radiation
μ = 0.12 mm^−1^
*T* = 100 K
0.30 × 0.20 × 0.20 mm

#### Data collection

Bruker Kappa APEXII DUO CCD diffractometer
Absorption correction: multi-scan (*SADABS*; Bruker, 2007 ▶) *T*_min_ = 0.86, *T*_max_ = 0.98
32220 measured reflections
6660 independent reflections
5882 reflections with *I* > 2σ(*I*)
*R*_int_ = 0.024

#### Refinement

*R*[*F* ^2^ > 2σ(*F* ^2^)] = 0.036
*wR*(*F* ^2^) = 0.089
*S* = 0.98
6660 reflections
469 parameters
H-atom parameters constrained
Δρ_max_ = 0.38 e Å^−3^
Δρ_min_ = −0.40 e Å^−3^

### 

Data collection: *APEX2* (Bruker, 2007 ▶); cell refinement: *SAINT* (Bruker, 2007 ▶); data reduction: *SAINT*; program(s) used to solve structure: *SHELXS97* (Sheldrick, 2008 ▶); program(s) used to refine structure: *SHELXL97* (Sheldrick, 2008 ▶); molecular graphics: *ORTEP-3 for Windows* (Farrugia, 1997 ▶), Mercury (Macrae *et al.*, 2008 ▶) and *OLEX2* (Dolomanov *et al.*, 2009 ▶).; software used to prepare material for publication: *publCIF* (Westrip, 2010 ▶).

## Supplementary Material

Crystal structure: contains datablock(s) global, I. DOI: 10.1107/S1600536812001675/nk2128sup1.cif

Structure factors: contains datablock(s) I. DOI: 10.1107/S1600536812001675/nk2128Isup2.hkl

Supplementary material file. DOI: 10.1107/S1600536812001675/nk2128Isup3.cml

Additional supplementary materials:  crystallographic information; 3D view; checkCIF report

## Figures and Tables

**Table 1:** Hydrogen-bond geometry (Å, °)

*D*—H⋯*A*	*D*—H	H⋯*A*	*D*⋯*A*	*D*—H⋯*A*
N3—H3⋯O1^i^	0.88	2.01	2.8395 (14)	156
N1—H1⋯O4	0.88	2.00	2.8757 (14)	173

Symmetry code: (i) 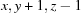.

**Fig. 1.**
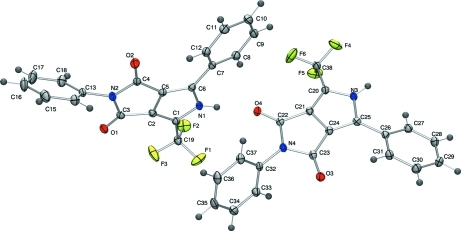
The two independent molecules in the asymmetric unit are shown. Anisotropically refined atoms are shown as 50% probability ellipsoids.

**Fig. 2.**
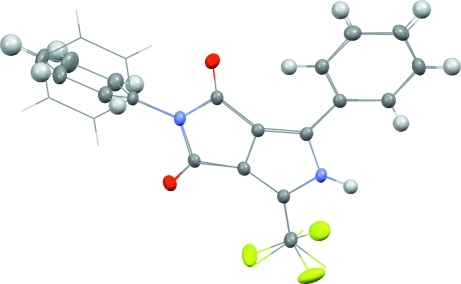
An overlay of the independent molecules in the asymmetric unit, one shown in a ball-and-stick representation, the other as wireframe. The only significant differences between the molecules are rotations of the phenyl and CF3 substituents.

**Fig. 3.**
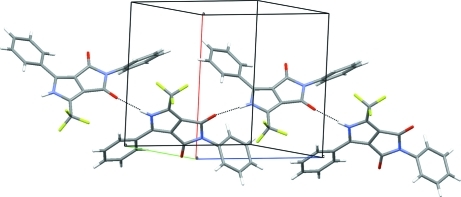
A view emphasizing chains of hydrogen bonded molecules. Hydrogen bonds are shown as blue dotted lines.

## supplementary crystallographic information

### Comment

The biological activity of compounds with pyrrol-3,4-dicarboximide scaffolds includes analgesic, central nervous system depressive action, and antiproliferative activities (Malinka *et al.* 2005; Malinka *et al.* 1999; Shen *et al.* 2010). Furthermore, pyrrol-3,4-dicarboximides are very interesting compounds because of their structural similarity to lamellarins (Yu *et al.* 2011). The title compound was synthesized and its crystal structure is reported herein.

The asymmetric unit contains two molecules of the title compound (see Figure 1 for a view of the molecular structure). After overlapping the central fused ring of the independent molecules using OLEX2 (see Figure 2; Dolomanov *et al.*, 2009), it is clear that the molecules differ only in rotation of the phenyl ring and CF_3_ substituents. The phenyl ring planes differ by approximately a 41° rotation about the N—C bond. The CF_3_ substituent is rotated by 16.5°. The r.m.s. deviation of atomic positions between the two molecules is 0.56 Å (all atoms), 0.065 Å for matched atoms. The center of one of the phenyl rings that differ in orientation (C7—C12) has a close contact (2.572 Å) to the hydrogen atom bonded to C14 on a symmetry related molecule. This contact is lacking for the other molecule.

Intermolecular hydrogen bonds connect molecules into a ribbon throughout the crystal. Figure 3 shows molecular packing and hydrogen bonds in the crystal. Hydrogen bonds exist between O1 and N3 (2.8395 Å) and O4 and N1 (2.8757 Å) and connect molecules into a chain normal to (0 1 - 1). Crystallographically unique molecules alternate along the hydrogen bonded chains. The graph set description is C2,2(12)>a>b (determined using Mercury (Macrae *et al.*, 2008)).

### Experimental

To a stirred solution of 4-phenyl-2-(trifluoromethyl) oxazol-5(4*H*)-one (0.3 g, 1.3 mmol) and 3-bromo-1-phenyl-1*H*-pyrrole-2,5-dione (0.33 g, 1.3 mmol) in toluene (20 mL), *N*,*N*-diisopropylethylamine (0.33 g, 0.45 ml, 2.6 mmol) was added at 298 K. The reaction mixture was stirred at room temperature for 15 minutes. Thereafter, the solvent was evaporated *in vacuo* and crude material purified by automated flash chromatography using a gradient from 100% Hexane to 70% Hexane/AcOEt. Crystals suitable for X-ray diffraction studies were obtained by recrystallization of the pure product from methylene chloride and hexane.

### Refinement

All hydrogen atoms were visible in a difference Fourier map and were added at calculated positions. Bonds distances are set to 0.95 Å for carbon-hydrogen bonds, and 0.88 Å for nitrogen-hydrogen bonds.

### Crystal data

**Table:**

C_19_H_11_F_3_N_2_O_2_	*Z* = 4
*M**_r_* = 356.30	*F*(000) = 728
Triclinic, *P*1	*D*_x_ = 1.512 Mg m^−^^3^
*a* = 10.4730 (4) Å	Mo *K*α radiation, λ = 0.71073 Å
*b* = 12.2394 (5) Å	Cell parameters from 9948 reflections
*c* = 13.4379 (5) Å	θ = 2.5–26.7°
α = 67.542 (2)°	µ = 0.12 mm^−^^1^
β = 82.511 (2)°	*T* = 100 K
γ = 80.294 (2)°	Prismatic, white
*V* = 1564.98 (11) Å^3^	0.30 × 0.20 × 0.20 mm

### Data collection

**Table:**

Bruker Kappa APEXII DUO CCD diffractometer	6660 independent reflections
Radiation source: fine-focus sealed tube	5882 reflections with *I* > 2σ(*I*)
graphite	*R*_int_ = 0.024
Detector resolution: 8.3333 pixels mm^-1^	θ_max_ = 26.8°, θ_min_ = 1.6°
φ and ω scans	*h* = −13→13
Absorption correction: multi-scan (*SADABS*; Bruker, 2007)	*k* = −15→15
*T*_min_ = 0.86, *T*_max_ = 0.98	*l* = −17→16
32220 measured reflections	

### Refinement

**Table:**

Refinement on *F*^2^	Primary atom site location: structure-invariant direct methods
Least-squares matrix: full	Secondary atom site location: difference Fourier map
*R*[*F*^2^ > 2σ(*F*^2^)] = 0.036	Hydrogen site location: inferred from neighbouring sites
*wR*(*F*^2^) = 0.089	H-atom parameters constrained
*S* = 0.98	*w* = 1/[σ^2^(*F*_o_^2^) + (0.0353*P*)^2^ + 1.1733*P*] where *P* = (*F*_o_^2^ + 2*F*_c_^2^)/3
6660 reflections	(Δ/σ)_max_ < 0.001
469 parameters	Δρ_max_ = 0.38 e Å^−^^3^
0 restraints	Δρ_min_ = −0.40 e Å^−^^3^

### Special details

**Table:**

Geometry. All e.s.d.'s (except the e.s.d. in the dihedral angle between two l.s. planes) are estimated using the full covariance matrix. The cell e.s.d.'s are taken into account individually in the estimation of e.s.d.'s in distances, angles and torsion angles; correlations between e.s.d.'s in cell parameters are only used when they are defined by crystal symmetry. An approximate (isotropic) treatment of cell e.s.d.'s is used for estimating e.s.d.'s involving l.s. planes.
Refinement. Refinement of *F*^2^ against ALL reflections. The weighted *R*-factor *wR* and goodness of fit *S* are based on *F*^2^, conventional *R*-factors *R* are based on *F*, with *F* set to zero for negative *F*^2^. The threshold expression of *F*^2^ > σ(*F*^2^) is used only for calculating *R*-factors(gt) *etc*. and is not relevant to the choice of reflections for refinement. *R*-factors based on *F*^2^ are statistically about twice as large as those based on *F*, and *R*- factors based on ALL data will be even larger.

### Fractional atomic coordinates and isotropic or equivalent isotropic displacement parameters (Å^2^)

**Table:**

	*x*	*y*	*z*	*U*_iso_*/*U*_eq_	
N1	0.35532 (11)	0.26068 (9)	0.56592 (9)	0.0148 (2)	
H1	0.325	0.3322	0.5223	0.018*	
N2	0.50857 (11)	−0.07836 (9)	0.78471 (9)	0.0160 (2)	
F2	0.21712 (9)	0.35605 (8)	0.72938 (8)	0.0317 (2)	
F3	0.14763 (10)	0.18726 (8)	0.81133 (8)	0.0427 (3)	
F1	0.08781 (9)	0.30162 (10)	0.65371 (8)	0.0374 (2)	
O1	0.34017 (9)	−0.03336 (8)	0.89917 (8)	0.0201 (2)	
O2	0.65695 (9)	−0.06867 (8)	0.63809 (8)	0.0202 (2)	
C12	0.63795 (13)	0.15446 (12)	0.41080 (11)	0.0177 (3)	
H12	0.6364	0.0713	0.4483	0.021*	
C1	0.30092 (12)	0.20234 (11)	0.66742 (10)	0.0152 (3)	
C6	0.46392 (12)	0.19190 (11)	0.54172 (10)	0.0143 (2)	
C3	0.39775 (12)	−0.00958 (11)	0.81056 (10)	0.0151 (3)	
C2	0.37467 (12)	0.09388 (11)	0.70964 (10)	0.0148 (2)	
C7	0.55052 (12)	0.23597 (11)	0.44434 (10)	0.0152 (3)	
C4	0.56091 (12)	−0.02501 (11)	0.67653 (10)	0.0152 (3)	
C5	0.47444 (12)	0.08653 (11)	0.63056 (10)	0.0141 (2)	
C13	0.56303 (13)	−0.19193 (11)	0.85754 (11)	0.0171 (3)	
C8	0.55377 (13)	0.35825 (12)	0.38800 (11)	0.0182 (3)	
H8	0.4948	0.4143	0.4099	0.022*	
C9	0.64284 (14)	0.39777 (13)	0.30042 (11)	0.0220 (3)	
H9	0.6443	0.4809	0.2623	0.026*	
C14	0.56688 (14)	−0.29193 (12)	0.83170 (12)	0.0214 (3)	
H14	0.5332	−0.2851	0.767	0.026*	
C11	0.72685 (14)	0.19496 (13)	0.32298 (11)	0.0218 (3)	
H11	0.7857	0.1394	0.3004	0.026*	
C18	0.61225 (15)	−0.20018 (13)	0.95143 (12)	0.0243 (3)	
H18	0.6099	−0.1312	0.9683	0.029*	
C10	0.72999 (14)	0.31659 (13)	0.26804 (11)	0.0232 (3)	
H10	0.7916	0.3441	0.2085	0.028*	
C15	0.62048 (15)	−0.40205 (13)	0.90134 (13)	0.0279 (3)	
H15	0.6235	−0.4711	0.8844	0.034*	
C16	0.66956 (16)	−0.41145 (14)	0.99549 (13)	0.0323 (4)	
H16	0.7063	−0.4869	1.043	0.039*	
C17	0.66517 (17)	−0.31135 (15)	1.02056 (12)	0.0326 (4)	
H17	0.6985	−0.3185	1.0855	0.039*	
C19	0.18769 (13)	0.26076 (12)	0.71546 (11)	0.0189 (3)	
F4	0.44762 (10)	0.84161 (10)	0.14618 (8)	0.0383 (3)	
F5	0.31060 (9)	0.84493 (9)	0.27591 (8)	0.0373 (2)	
F6	0.44468 (11)	0.68802 (9)	0.29453 (10)	0.0485 (3)	
O4	0.25551 (9)	0.48629 (8)	0.41050 (7)	0.0179 (2)	
O3	−0.02763 (9)	0.49810 (8)	0.17043 (8)	0.0192 (2)	
N3	0.23046 (11)	0.79102 (10)	0.08260 (9)	0.0165 (2)	
H3	0.2561	0.8585	0.0371	0.02*	
N4	0.10361 (11)	0.46869 (9)	0.30785 (9)	0.0154 (2)	
C22	0.19988 (12)	0.52244 (11)	0.32763 (10)	0.0147 (2)	
C26	0.06391 (12)	0.78966 (12)	−0.03297 (10)	0.0157 (3)	
C25	0.13700 (12)	0.73589 (11)	0.06384 (10)	0.0151 (3)	
C21	0.21500 (12)	0.62799 (11)	0.22789 (10)	0.0152 (3)	
C23	0.05479 (12)	0.53067 (11)	0.20333 (10)	0.0151 (3)	
C24	0.12802 (12)	0.63298 (11)	0.15423 (10)	0.0150 (3)	
C20	0.27846 (13)	0.72673 (12)	0.18161 (11)	0.0168 (3)	
C31	0.00339 (14)	0.71767 (12)	−0.06663 (11)	0.0190 (3)	
H31	0.0126	0.6338	−0.0286	0.023*	
C32	0.05449 (13)	0.36364 (11)	0.38542 (10)	0.0157 (3)	
C28	−0.02495 (14)	0.96323 (12)	−0.17831 (11)	0.0217 (3)	
H28	−0.0347	1.0471	−0.2165	0.026*	
C27	0.04938 (13)	0.91299 (12)	−0.08956 (11)	0.0187 (3)	
H27	0.0905	0.9625	−0.0672	0.022*	
C37	0.13017 (14)	0.25396 (12)	0.40797 (11)	0.0193 (3)	
H37	0.2151	0.248	0.3739	0.023*	
C36	0.08002 (15)	0.15257 (12)	0.48124 (12)	0.0236 (3)	
H36	0.1313	0.0768	0.4983	0.028*	
C30	−0.07021 (14)	0.76880 (13)	−0.15566 (12)	0.0224 (3)	
H30	−0.1108	0.7196	−0.1787	0.027*	
C34	−0.11889 (14)	0.27239 (13)	0.50622 (12)	0.0242 (3)	
H34	−0.2041	0.2783	0.5397	0.029*	
C33	−0.06943 (14)	0.37408 (12)	0.43446 (11)	0.0206 (3)	
H33	−0.1198	0.4501	0.419	0.025*	
C29	−0.08508 (14)	0.89119 (13)	−0.21134 (11)	0.0222 (3)	
H29	−0.1362	0.9257	−0.2719	0.027*	
C38	0.37168 (14)	0.77383 (12)	0.22422 (11)	0.0199 (3)	
C35	−0.04424 (15)	0.16186 (13)	0.52922 (11)	0.0245 (3)	
H35	−0.0787	0.0922	0.5782	0.029*	

### Atomic displacement parameters (Å^2^)

**Table:**

	*U*^11^	*U*^22^	*U*^33^	*U*^12^	*U*^13^	*U*^23^
N1	0.0173 (5)	0.0114 (5)	0.0137 (5)	−0.0008 (4)	−0.0019 (4)	−0.0026 (4)
N2	0.0183 (5)	0.0130 (5)	0.0143 (5)	−0.0014 (4)	−0.0012 (4)	−0.0026 (4)
F2	0.0338 (5)	0.0266 (5)	0.0431 (6)	−0.0007 (4)	−0.0007 (4)	−0.0242 (4)
F3	0.0475 (6)	0.0241 (5)	0.0342 (5)	0.0060 (4)	0.0236 (5)	0.0010 (4)
F1	0.0219 (5)	0.0552 (6)	0.0438 (6)	0.0131 (4)	−0.0119 (4)	−0.0324 (5)
O1	0.0212 (5)	0.0188 (5)	0.0164 (5)	−0.0041 (4)	0.0026 (4)	−0.0030 (4)
O2	0.0202 (5)	0.0185 (5)	0.0189 (5)	0.0020 (4)	0.0005 (4)	−0.0061 (4)
C12	0.0195 (6)	0.0172 (6)	0.0164 (6)	−0.0021 (5)	−0.0031 (5)	−0.0055 (5)
C1	0.0164 (6)	0.0139 (6)	0.0149 (6)	−0.0025 (5)	−0.0018 (5)	−0.0044 (5)
C6	0.0151 (6)	0.0142 (6)	0.0147 (6)	−0.0015 (5)	−0.0037 (5)	−0.0058 (5)
C3	0.0160 (6)	0.0132 (6)	0.0167 (6)	−0.0043 (5)	−0.0012 (5)	−0.0053 (5)
C2	0.0158 (6)	0.0143 (6)	0.0150 (6)	−0.0035 (5)	−0.0008 (5)	−0.0057 (5)
C7	0.0157 (6)	0.0171 (6)	0.0129 (6)	−0.0035 (5)	−0.0027 (5)	−0.0047 (5)
C4	0.0172 (6)	0.0140 (6)	0.0146 (6)	−0.0032 (5)	−0.0028 (5)	−0.0045 (5)
C5	0.0148 (6)	0.0141 (6)	0.0142 (6)	−0.0024 (5)	−0.0018 (5)	−0.0058 (5)
C13	0.0170 (6)	0.0139 (6)	0.0153 (6)	−0.0010 (5)	−0.0004 (5)	−0.0003 (5)
C8	0.0195 (6)	0.0171 (6)	0.0182 (6)	−0.0026 (5)	−0.0024 (5)	−0.0063 (5)
C9	0.0243 (7)	0.0197 (7)	0.0192 (7)	−0.0074 (5)	−0.0024 (5)	−0.0022 (5)
C14	0.0233 (7)	0.0179 (7)	0.0211 (7)	−0.0019 (5)	−0.0024 (5)	−0.0052 (6)
C11	0.0193 (7)	0.0268 (7)	0.0190 (7)	−0.0002 (5)	−0.0001 (5)	−0.0096 (6)
C18	0.0283 (8)	0.0243 (7)	0.0186 (7)	−0.0010 (6)	−0.0029 (6)	−0.0067 (6)
C10	0.0207 (7)	0.0296 (8)	0.0161 (7)	−0.0070 (6)	0.0021 (5)	−0.0044 (6)
C15	0.0309 (8)	0.0157 (7)	0.0307 (8)	0.0000 (6)	0.0024 (6)	−0.0042 (6)
C16	0.0350 (9)	0.0228 (8)	0.0229 (8)	0.0068 (6)	−0.0013 (6)	0.0047 (6)
C17	0.0379 (9)	0.0355 (9)	0.0163 (7)	0.0030 (7)	−0.0077 (6)	−0.0020 (6)
C19	0.0212 (7)	0.0153 (6)	0.0180 (6)	−0.0004 (5)	−0.0002 (5)	−0.0049 (5)
F4	0.0402 (6)	0.0568 (6)	0.0266 (5)	−0.0341 (5)	0.0090 (4)	−0.0172 (5)
F5	0.0337 (5)	0.0514 (6)	0.0435 (6)	−0.0149 (4)	0.0028 (4)	−0.0339 (5)
F6	0.0552 (7)	0.0263 (5)	0.0633 (7)	−0.0056 (5)	−0.0444 (6)	−0.0029 (5)
O4	0.0207 (5)	0.0152 (4)	0.0159 (5)	−0.0006 (4)	−0.0047 (4)	−0.0032 (4)
O3	0.0220 (5)	0.0173 (5)	0.0189 (5)	−0.0057 (4)	−0.0032 (4)	−0.0056 (4)
N3	0.0199 (6)	0.0138 (5)	0.0142 (5)	−0.0054 (4)	−0.0022 (4)	−0.0018 (4)
N4	0.0181 (5)	0.0121 (5)	0.0143 (5)	−0.0025 (4)	−0.0014 (4)	−0.0027 (4)
C22	0.0156 (6)	0.0127 (6)	0.0153 (6)	0.0001 (5)	0.0002 (5)	−0.0058 (5)
C26	0.0159 (6)	0.0173 (6)	0.0121 (6)	−0.0017 (5)	0.0002 (5)	−0.0041 (5)
C25	0.0164 (6)	0.0138 (6)	0.0155 (6)	−0.0021 (5)	0.0002 (5)	−0.0062 (5)
C21	0.0158 (6)	0.0142 (6)	0.0148 (6)	−0.0009 (5)	−0.0008 (5)	−0.0048 (5)
C23	0.0168 (6)	0.0123 (6)	0.0148 (6)	0.0004 (5)	−0.0001 (5)	−0.0049 (5)
C24	0.0158 (6)	0.0141 (6)	0.0145 (6)	−0.0010 (5)	−0.0010 (5)	−0.0053 (5)
C20	0.0184 (6)	0.0161 (6)	0.0148 (6)	−0.0026 (5)	−0.0017 (5)	−0.0040 (5)
C31	0.0239 (7)	0.0163 (6)	0.0165 (6)	−0.0037 (5)	−0.0021 (5)	−0.0049 (5)
C32	0.0211 (6)	0.0129 (6)	0.0123 (6)	−0.0042 (5)	−0.0024 (5)	−0.0027 (5)
C28	0.0240 (7)	0.0166 (6)	0.0201 (7)	−0.0017 (5)	−0.0042 (5)	−0.0014 (5)
C27	0.0205 (7)	0.0176 (6)	0.0178 (6)	−0.0038 (5)	−0.0019 (5)	−0.0057 (5)
C37	0.0240 (7)	0.0159 (6)	0.0177 (6)	−0.0008 (5)	−0.0018 (5)	−0.0064 (5)
C36	0.0369 (8)	0.0127 (6)	0.0201 (7)	−0.0017 (6)	−0.0052 (6)	−0.0044 (5)
C30	0.0255 (7)	0.0239 (7)	0.0203 (7)	−0.0059 (6)	−0.0048 (6)	−0.0087 (6)
C34	0.0221 (7)	0.0286 (8)	0.0188 (7)	−0.0081 (6)	0.0004 (5)	−0.0039 (6)
C33	0.0210 (7)	0.0176 (6)	0.0193 (7)	−0.0008 (5)	−0.0018 (5)	−0.0033 (5)
C29	0.0218 (7)	0.0252 (7)	0.0174 (7)	−0.0027 (6)	−0.0062 (5)	−0.0039 (6)
C38	0.0218 (7)	0.0193 (7)	0.0173 (7)	−0.0067 (5)	−0.0031 (5)	−0.0033 (5)
C35	0.0368 (8)	0.0197 (7)	0.0160 (7)	−0.0138 (6)	−0.0029 (6)	−0.0010 (5)

### Geometric parameters (Å, °)

**Table:**

N1—C1	1.3731 (16)	F4—C38	1.3269 (16)
N1—C6	1.3776 (16)	F5—C38	1.3444 (17)
N1—H1	0.8800	F6—C38	1.3190 (17)
N2—C3	1.4018 (17)	O4—C22	1.2170 (16)
N2—C4	1.4240 (16)	O3—C23	1.2040 (16)
N2—C13	1.4332 (16)	N3—C20	1.3723 (17)
F2—C19	1.3391 (16)	N3—C25	1.3761 (17)
F3—C19	1.3213 (16)	N3—H3	0.8800
F1—C19	1.3288 (17)	N4—C22	1.3954 (17)
O1—C3	1.2139 (16)	N4—C23	1.4324 (16)
O2—C4	1.2076 (16)	N4—C32	1.4366 (16)
C12—C11	1.3884 (19)	C22—C21	1.4769 (17)
C12—C7	1.4018 (18)	C26—C31	1.3964 (19)
C12—H12	0.9500	C26—C27	1.3985 (18)
C1—C2	1.3689 (18)	C26—C25	1.4654 (18)
C1—C19	1.4837 (18)	C25—C24	1.3827 (18)
C6—C5	1.3823 (18)	C21—C20	1.3704 (18)
C6—C7	1.4636 (18)	C21—C24	1.4083 (18)
C3—C2	1.4735 (18)	C23—C24	1.4670 (18)
C2—C5	1.4074 (18)	C20—C38	1.4835 (18)
C7—C8	1.3996 (18)	C31—C30	1.3867 (19)
C4—C5	1.4663 (17)	C31—H31	0.9500
C13—C18	1.386 (2)	C32—C37	1.3840 (18)
C13—C14	1.3873 (19)	C32—C33	1.3858 (19)
C8—C9	1.3858 (19)	C28—C27	1.3881 (19)
C8—H8	0.9500	C28—C29	1.389 (2)
C9—C10	1.389 (2)	C28—H28	0.9500
C9—H9	0.9500	C27—H27	0.9500
C14—C15	1.388 (2)	C37—C36	1.3917 (19)
C14—H14	0.9500	C37—H37	0.9500
C11—C10	1.390 (2)	C36—C35	1.382 (2)
C11—H11	0.9500	C36—H36	0.9500
C18—C17	1.391 (2)	C30—C29	1.387 (2)
C18—H18	0.9500	C30—H30	0.9500
C10—H10	0.9500	C34—C33	1.3847 (19)
C15—C16	1.384 (2)	C34—C35	1.387 (2)
C15—H15	0.9500	C34—H34	0.9500
C16—C17	1.382 (2)	C33—H33	0.9500
C16—H16	0.9500	C29—H29	0.9500
C17—H17	0.9500	C35—H35	0.9500
			
C1—N1—C6	110.75 (10)	C20—N3—C25	111.02 (11)
C1—N1—H1	124.6	C20—N3—H3	124.5
C6—N1—H1	124.6	C25—N3—H3	124.5
C3—N2—C4	112.92 (10)	C22—N4—C23	113.47 (10)
C3—N2—C13	124.26 (11)	C22—N4—C32	123.71 (11)
C4—N2—C13	122.80 (11)	C23—N4—C32	122.80 (11)
C11—C12—C7	120.19 (12)	O4—C22—N4	125.38 (12)
C11—C12—H12	119.9	O4—C22—C21	130.17 (12)
C7—C12—H12	119.9	N4—C22—C21	104.44 (11)
C2—C1—N1	107.49 (11)	C31—C26—C27	119.49 (12)
C2—C1—C19	131.11 (12)	C31—C26—C25	119.70 (12)
N1—C1—C19	121.27 (11)	C27—C26—C25	120.75 (12)
N1—C6—C5	105.48 (11)	N3—C25—C24	105.57 (11)
N1—C6—C7	123.03 (11)	N3—C25—C26	122.20 (11)
C5—C6—C7	131.18 (12)	C24—C25—C26	132.11 (12)
O1—C3—N2	125.03 (12)	C20—C21—C24	107.44 (11)
O1—C3—C2	130.37 (12)	C20—C21—C22	143.29 (12)
N2—C3—C2	104.56 (10)	C24—C21—C22	109.19 (11)
C1—C2—C5	107.14 (11)	O3—C23—N4	123.71 (12)
C1—C2—C3	142.97 (12)	O3—C23—C24	132.10 (12)
C5—C2—C3	109.22 (11)	N4—C23—C24	104.19 (10)
C8—C7—C12	119.19 (12)	C25—C24—C21	108.83 (11)
C8—C7—C6	121.21 (12)	C25—C24—C23	142.33 (12)
C12—C7—C6	119.47 (12)	C21—C24—C23	108.72 (11)
O2—C4—N2	123.74 (12)	C21—C20—N3	107.14 (11)
O2—C4—C5	131.42 (12)	C21—C20—C38	131.64 (12)
N2—C4—C5	104.84 (11)	N3—C20—C38	120.92 (11)
C6—C5—C2	109.10 (11)	C30—C31—C26	119.88 (12)
C6—C5—C4	142.06 (12)	C30—C31—H31	120.1
C2—C5—C4	108.36 (11)	C26—C31—H31	120.1
C18—C13—C14	121.11 (13)	C37—C32—C33	121.29 (12)
C18—C13—N2	119.90 (12)	C37—C32—N4	119.62 (12)
C14—C13—N2	118.98 (12)	C33—C32—N4	119.07 (12)
C9—C8—C7	120.18 (13)	C27—C28—C29	120.13 (13)
C9—C8—H8	119.9	C27—C28—H28	119.9
C7—C8—H8	119.9	C29—C28—H28	119.9
C8—C9—C10	120.40 (13)	C28—C27—C26	120.12 (13)
C8—C9—H9	119.8	C28—C27—H27	119.9
C10—C9—H9	119.8	C26—C27—H27	119.9
C13—C14—C15	119.34 (14)	C32—C37—C36	118.99 (13)
C13—C14—H14	120.3	C32—C37—H37	120.5
C15—C14—H14	120.3	C36—C37—H37	120.5
C12—C11—C10	120.19 (13)	C35—C36—C37	120.13 (13)
C12—C11—H11	119.9	C35—C36—H36	119.9
C10—C11—H11	119.9	C37—C36—H36	119.9
C13—C18—C17	118.80 (14)	C31—C30—C29	120.55 (13)
C13—C18—H18	120.6	C31—C30—H30	119.7
C17—C18—H18	120.6	C29—C30—H30	119.7
C9—C10—C11	119.85 (13)	C33—C34—C35	120.09 (14)
C9—C10—H10	120.1	C33—C34—H34	120.0
C11—C10—H10	120.1	C35—C34—H34	120.0
C16—C15—C14	120.06 (15)	C34—C33—C32	119.20 (13)
C16—C15—H15	120.0	C34—C33—H33	120.4
C14—C15—H15	120.0	C32—C33—H33	120.4
C17—C16—C15	120.17 (14)	C30—C29—C28	119.82 (13)
C17—C16—H16	119.9	C30—C29—H29	120.1
C15—C16—H16	119.9	C28—C29—H29	120.1
C16—C17—C18	120.51 (15)	F6—C38—F4	109.15 (12)
C16—C17—H17	119.7	F6—C38—F5	106.22 (12)
C18—C17—H17	119.7	F4—C38—F5	104.84 (11)
F3—C19—F1	108.45 (12)	F6—C38—C20	112.20 (11)
F3—C19—F2	106.94 (12)	F4—C38—C20	112.23 (11)
F1—C19—F2	105.21 (11)	F5—C38—C20	111.78 (11)
F3—C19—C1	111.26 (11)	C36—C35—C34	120.29 (13)
F1—C19—C1	113.09 (11)	C36—C35—H35	119.9
F2—C19—C1	111.52 (11)	C34—C35—H35	119.9
			
C6—N1—C1—C2	−0.33 (14)	C23—N4—C22—O4	179.89 (12)
C6—N1—C1—C19	175.96 (11)	C32—N4—C22—O4	1.8 (2)
C1—N1—C6—C5	1.50 (14)	C23—N4—C22—C21	−0.01 (14)
C1—N1—C6—C7	−172.82 (11)	C32—N4—C22—C21	−178.08 (11)
C4—N2—C3—O1	−175.96 (12)	C20—N3—C25—C24	−0.67 (15)
C13—N2—C3—O1	5.5 (2)	C20—N3—C25—C26	175.96 (12)
C4—N2—C3—C2	2.17 (14)	C31—C26—C25—N3	159.02 (12)
C13—N2—C3—C2	−176.41 (11)	C27—C26—C25—N3	−23.69 (19)
N1—C1—C2—C5	−0.97 (14)	C31—C26—C25—C24	−25.4 (2)
C19—C1—C2—C5	−176.76 (13)	C27—C26—C25—C24	151.93 (14)
N1—C1—C2—C3	167.80 (16)	O4—C22—C21—C20	−3.9 (3)
C19—C1—C2—C3	−8.0 (3)	N4—C22—C21—C20	176.03 (18)
O1—C3—C2—C1	6.3 (3)	O4—C22—C21—C24	−179.59 (13)
N2—C3—C2—C1	−171.73 (17)	N4—C22—C21—C24	0.30 (14)
O1—C3—C2—C5	174.89 (13)	C22—N4—C23—O3	179.29 (12)
N2—C3—C2—C5	−3.10 (14)	C32—N4—C23—O3	−2.62 (19)
C11—C12—C7—C8	0.25 (19)	C22—N4—C23—C24	−0.26 (14)
C11—C12—C7—C6	−175.56 (12)	C32—N4—C23—C24	177.83 (11)
N1—C6—C7—C8	20.70 (19)	N3—C25—C24—C21	1.02 (14)
C5—C6—C7—C8	−152.02 (14)	C26—C25—C24—C21	−175.14 (13)
N1—C6—C7—C12	−163.58 (12)	N3—C25—C24—C23	176.03 (16)
C5—C6—C7—C12	23.7 (2)	C26—C25—C24—C23	−0.1 (3)
C3—N2—C4—O2	179.17 (12)	C20—C21—C24—C25	−1.01 (15)
C13—N2—C4—O2	−2.2 (2)	C22—C21—C24—C25	176.32 (11)
C3—N2—C4—C5	−0.48 (14)	C20—C21—C24—C23	−177.80 (11)
C13—N2—C4—C5	178.13 (11)	C22—C21—C24—C23	−0.47 (14)
N1—C6—C5—C2	−2.08 (14)	O3—C23—C24—C25	5.9 (3)
C7—C6—C5—C2	171.59 (13)	N4—C23—C24—C25	−174.58 (17)
N1—C6—C5—C4	−172.55 (16)	O3—C23—C24—C21	−179.05 (14)
C7—C6—C5—C4	1.1 (3)	N4—C23—C24—C21	0.44 (13)
C1—C2—C5—C6	1.93 (15)	C24—C21—C20—N3	0.58 (15)
C3—C2—C5—C6	−170.94 (11)	C22—C21—C20—N3	−175.20 (17)
C1—C2—C5—C4	175.77 (11)	C24—C21—C20—C38	174.17 (14)
C3—C2—C5—C4	2.91 (14)	C22—C21—C20—C38	−1.6 (3)
O2—C4—C5—C6	−10.6 (3)	C25—N3—C20—C21	0.06 (15)
N2—C4—C5—C6	168.98 (16)	C25—N3—C20—C38	−174.36 (12)
O2—C4—C5—C2	178.85 (14)	C27—C26—C31—C30	0.1 (2)
N2—C4—C5—C2	−1.54 (13)	C25—C26—C31—C30	177.41 (13)
C3—N2—C13—C18	−63.64 (18)	C22—N4—C32—C37	−73.65 (17)
C4—N2—C13—C18	117.91 (15)	C23—N4—C32—C37	108.45 (14)
C3—N2—C13—C14	117.16 (15)	C22—N4—C32—C33	107.80 (15)
C4—N2—C13—C14	−61.29 (17)	C23—N4—C32—C33	−70.09 (17)
C12—C7—C8—C9	−0.22 (19)	C29—C28—C27—C26	−0.1 (2)
C6—C7—C8—C9	175.51 (12)	C31—C26—C27—C28	0.2 (2)
C7—C8—C9—C10	−0.3 (2)	C25—C26—C27—C28	−177.12 (12)
C18—C13—C14—C15	0.3 (2)	C33—C32—C37—C36	0.1 (2)
N2—C13—C14—C15	179.44 (13)	N4—C32—C37—C36	−178.38 (12)
C7—C12—C11—C10	0.2 (2)	C32—C37—C36—C35	0.9 (2)
C14—C13—C18—C17	−0.5 (2)	C26—C31—C30—C29	−0.4 (2)
N2—C13—C18—C17	−179.69 (13)	C35—C34—C33—C32	0.7 (2)
C12—C11—C10—C9	−0.7 (2)	C37—C32—C33—C34	−0.9 (2)
C8—C9—C10—C11	0.8 (2)	N4—C32—C33—C34	177.59 (12)
C13—C14—C15—C16	0.0 (2)	C31—C30—C29—C28	0.5 (2)
C14—C15—C16—C17	0.1 (2)	C27—C28—C29—C30	−0.3 (2)
C15—C16—C17—C18	−0.4 (3)	C21—C20—C38—F6	32.2 (2)
C13—C18—C17—C16	0.6 (2)	N3—C20—C38—F6	−154.97 (13)
C2—C1—C19—F3	−7.4 (2)	C21—C20—C38—F4	155.50 (14)
N1—C1—C19—F3	177.30 (12)	N3—C20—C38—F4	−31.64 (18)
C2—C1—C19—F1	−129.75 (15)	C21—C20—C38—F5	−87.03 (18)
N1—C1—C19—F1	54.95 (17)	N3—C20—C38—F5	85.83 (15)
C2—C1—C19—F2	111.91 (16)	C37—C36—C35—C34	−1.2 (2)
N1—C1—C19—F2	−63.39 (16)	C33—C34—C35—C36	0.4 (2)

### Hydrogen-bond geometry (Å, °)

**Table:**

*D*—H···*A*	*D*—H	H···*A*	*D*···*A*	*D*—H···*A*
N3—H3···O1^i^	0.88	2.01	2.8395 (14)	156.
N1—H1···O4	0.88	2.00	2.8757 (14)	173.

Symmetry codes: (i) *x*, *y*+1, *z*−1.
